# Recent Progress in Self-Healable Hydrogel-Based Electroluminescent Devices: A Comprehensive Review

**DOI:** 10.3390/gels9030250

**Published:** 2023-03-21

**Authors:** Melkie Getnet Tadesse, Jörn Felix Lübben

**Affiliations:** 1 Sustainable Engineering (STE), Albstadt-Sigmaringen University, 72458 Albstadt, Germany; 2 Ethiopian Institute of Textile and Fashion Technology, Bahir Dar University, Bahir Dar 1037, Ethiopia

**Keywords:** conductive hydrogel, electroluminescence, flexible electronics, light-emitting diodes, conductive polymer, self-healing

## Abstract

Flexible electronics have gained significant research attention in recent years due to their potential applications as smart and functional materials. Typically, electroluminescence devices produced by hydrogel-based materials are among the most notable flexible electronics. With their excellent flexibility and their remarkable electrical, adaptable mechanical and self-healing properties, functional hydrogels offer a wealth of insights and opportunities for the fabrication of electroluminescent devices that can be easily integrated into wearable electronics for various applications. Various strategies have been developed and adapted to obtain functional hydrogels, and at the same time, high-performance electroluminescent devices have been fabricated based on these functional hydrogels. This review provides a comprehensive overview of various functional hydrogels that have been used for the development of electroluminescent devices. It also highlights some challenges and future research prospects for hydrogel-based electroluminescent devices.

## 1. Introduction

The development of flexible electronics has increased very rapidly in recent years [1]. This is because most conventional production methods for functional and technical flexible electronics and textiles have difficulties in terms of thermal [2] and tactile comfort during operation and use. Textile-based electronics are preferred due to their comfort properties [3]. Electroluminescence (EL) is exhibited by one type of electronic material that must be flexible, stretchable, soft and pliable to be used in wearable systems.

Over the years, hydrogels have played an important role in the design of flexible electronics. Individual cross-linkable polymers can be combined to form three-dimensional materials called hydrogels; the cross-linking of polymers can be either physical or chemical. Lim and Wichterle originally described these hydrogels in 1960 [4]. Flexible and wearable electronics have recently attracted much attention for several reasons, such as their ease of processing, e.g., by coating [5] and (three-dimensional) 3D printing [6]; lightweight and ductile properties [7]; excellent electromechanical properties [8]; and good integrability with clothing materials [9]. Flexible electronics have been explored for several applications, including flexible circuits, flexible displays, flexible solar cells, skin-like pressure sensors and conformable radio-frequency identification (RFID) tags [10]. In addition, flexible electronics have found diverse applications in health monitoring [11], emotion detection [12], electroluminescence device fabrication [13], supercapacitors [14,15] and so on. Inspiration from these diverse applications of flexible electronics and the extra-ordinary properties of hydrogels have made the development of functional and smart materials, such as the fabrication of electroluminescent devices, became a hot topic again [16].

The rapid development of advanced light-emitting diodes (LEDs) promotes today’s use of the phenomenon of flexible electronics world for future development. People are always demanding simple and convenient procedures in every aspect of life. Electroluminescent devices or light-emitting diodes (LEDs) are cutting-edge optical-electrical products that have gained enormous attention in recent decades [17,18] after it was originally discovered by Destriau in 1936 [19]. In this discovery, light emissions from a ZnS phosphor powder were detected. In this context, hydrogels have an important ability to respond to external stimuli, such as light, which is mentioned in [20].

**Figure 1:**
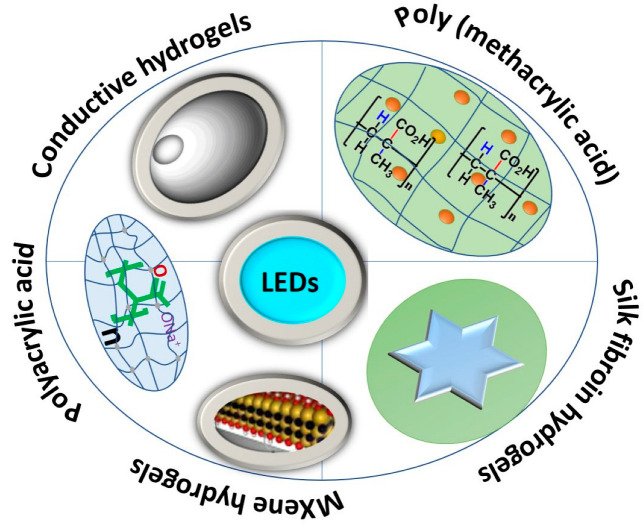
Different materials for hydrogel-based electroluminescent applications.

The main objective of this review article is to summarize the applications of hydrogels for electroluminescence device fabrication as shown in Figure 1. Hydrogels used for electroluminescence fabrication can be obtained from silk fibroin, polyacrylic acid, poly (methacrylic acid), conductive materials and MXene. These are the most used sources of functional hydrogels for the fabrication of self-healing electroluminescent devices. This overview discusses various aspects and circumstances of hydrogels that can be used to fabricate flexible electroluminescent displays (ELDs) and El. Furthermore, this review ends by incorporating findings from hydrogels with certain challenges, prospects and various strategies to mitigate the shortcomings of future LEDs in light-emitting applications.

The EL device can be constructed using two approaches, namely bottom-emission or top-emission structures. The most frequently used substrates to manufacture flexible electroluminescence materials are textile-based or film-based materials. For instance, in Ref. [21], the authors described a top-emission structure and Ag nanowires serving as both top and bottom electrodes. The results showed perfect flexibility and stretchability (88.4% luminance after 1000 bending–recovery cycles) and high luminance (957 cd m^−2^ @ 195 V and 2 kHz). Similarly, printable inorganic alternating-current electroluminescence devices with a bottom-emission structure (BES), top-emission structure (TES) and planar electrode structure (PES) were effectively implemented and reported [22]. By this, a deeper blue color observed. Hence, both bottom and top emissions with various substates are available options in EL device fabrication.

## 2. Introduction to Electroluminescence Devices 

**Figure 2:**
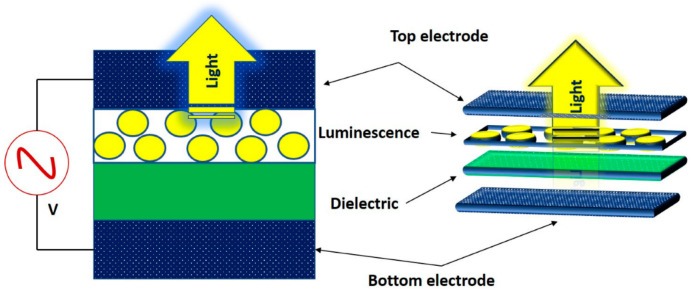
Basic structure of electroluminescence device.

Over many recent decades, researchers have been developing and discovering numerous flexible electronics, including electroluminescence devices and other soft electronics. Captain Henry Round discovered electroluminescence (EL) as observed in silicon carbide (SiC) in 1907 [23]. The yellow light observed when an electric current passes through a silicon carbide detector was the breakthrough that yielded today’s electroluminescence development. By definition, EL can be demarcated as the generation of different colored light when a current passes through light-emitting pastes, such as phosphor and zinc-based compounds. The source of light is from a layer of electroluminescent phosphor activated by an electric field. The electric field is applied between two parallel electrodes (lower and upper or negative and positive electrode) (Figure 2). The upper electrode should be transparent so that light can pass through. Dynamic developments being made in the electroluminescence field and universal applications of light-emitting diodes are becoming superficial. The basic electrode arrangement for electroluminescence device is lower electrode→separator→luminescence→upper electrode. To protect the upper electrode from environmental degradation, microencapsulation should occur, and the microcapsule has to be made of transparent materials. Furthermore, capsule materials are being employed to protect short circuits. Electroluminescence devices require a low consumption of power compared to other electronics. Light was emitted with 12 V alternating-current (AC) power source [6]. 

Although certain modifications are possible, the basic structure of the EL device is illustrated in Figure 2.

The principle behind the design of any display mechanism lies in the electroluminescent devices. Practical applications include fashion shows and talent shows, such as Britain’s Got Talent (BGT) and America’s Got Talent (AGT), as well as LEDs and car displays, to name a few examples. Electroluminescence can be fabricated from various types of electrode materials, such as conjugated polymers [24], pyrazoline derivatives [25], organic materials [26], hyperbranched polymers [27], spiro compounds [28], polysilane molecules [29] and various conductive polymers [30,31,32,33,34,35,36]. Among the conductive polymers, poly (3,4-ethylenedioxythiophene): poly (styrenesulfonate) (PEDOT:PSS) has emerged as a promising candidate for renewable, clean and reliable electroluminescence materials for the construction of lower and top electrodes [6,37,38,39,40,41,42]. However, in this review, we only focused on self-healable hydrogel materials for electroluminescence applications.

## 3. EL Devices Based on Self-Healable Hydrogels

Self-healing generally refers to a process of recovery after failures caused by mechanical pressures. The lifetime of a conventional EL device is restricted due to the damage that occurs when mechanical strain exceeds the resistance limit. Therefore, current developments of EL devices focus on developing electrode materials that can recover after stress, so that the lifetime and reliability of the EL device improve. Such device construction requires self-healable materials. Hydrogel-based materials are perfect candidates for this type of application. In this section, details of self-healing hydrogels and their synthesis mechanisms are discussed in detail.

### 3.1. Self-Healable Hydrogels

Electroluminescent devices have been sufficiently studied and realized using various materials, such as the light-emitting element. However, the durability of electroluminescent devices remains a challenge as abrasion and mechanical stress lead to the deterioration of the light-emitting element [43]. This paper presents various hydrogels with self-healing properties that can withstand mechanical stresses during operation and use. Nature plays an important role in the development of functional materials. Many new discoveries of today’s functional materials were inspired by nature [44]. For example, inspired by the luminosity of insects, the properties of bioluminescence were discovered as reported in [45]. Such inspirations help us to develop today’s electroluminescence technology by integrating model concepts into the design. Qian, X. et al. [46] developed self-healing flexible perovskite light-emitting diodes (PeLEDs) from a biologically inspired pangolin design. Developed PeLEDs have shown better bending strength. Because most wearable LEDs are subject to bending and twisting during wear, these resistance properties can be restored. 

Other nature-inspired materials for the applications of self-healing light-emitting diodes include human-skin-inspired electrospun electronic skins [47] and natural nano-clay-based [48] and cytoskeleton-inspired hydrogels [49]. At the rear end, when such kinds of resistance are not garneted, cracks can occur, which reduces the electrical characteristics of the materials that are the main components of LEDs. It is common practice to measure efficiency against bending and twisting for several cycles to check the durability of such materials. The first electroluminescence device was exposed to loss of its properties due to mechanical pressures. Scientists have tried to eliminate these kinds of challenges, again by nature inspiration, with self-healing properties, i.e., the process of recovery in which the device can repair oneself itself. This phenomena is based on natural biological systems where researchers used this concept and adapted it to create self-healable functional materials [50]. Therefore, self-healing means the materials can restore their physical and mechanical, as well as structural, properties after being deformed by external stimuli, such as strain [51]. This phenomenon is typically very important for electroluminescence devices as it can be subjected to several external stimuli, such as light, electric, bending, stress and so on [52]. Moreover, nature-inspired hydrogel materials and their designs have also been explored to produce sustainable and low-cost electroluminescence devices.

Self-healable gels are most often fitted in wearable textiles [53]. Most electroluminescence devices can be integrated into wearable textiles, which are subjected to several instances of bending, abrasion, load, and tightening during wear [52]. Another important parameter for wearable electronics is their stretchability, which plays a crucial role in developing flexible electroluminescence devices. In this aspect, several methods have been employed to enhance the stretchability of electroluminescence electrodes. Among them, are self-healing and self-bonding, and with them a high-strength polyurethane-based EL device has been fabricated [54]. EL devices showed excellent performance against stretchability. Polyurethane (PU) is a highly stretchable material [55]. In addition, PU exhibited the enhancement of stretchability in EL devices, which is very useful for wearable devices. More importantly, the EL device is feasible in terms of applied load, water and other external forces due to dynamic bonding and the stretchability of PU. Tiwari and Mathews [56] reported the PU derivative based on the Diels–Alder (DA) chemistry (PU-DA) self-healable polymeric composite for EL applications. At a rapid low-temperature range, the PU-DA composite unveiled extremely attractive dielectric properties, and the dielectric constant improved from 2.7 to 12.9. This was due to the highly flexible and stretchable nature of polyurethane materials. This proved it to be a self-healable and highly flexible material that is feasible for the fabrication of EL devices with improved physicomechanical properties. Cho, S.H. et al. [57] demonstrated another very important finding with regard to deformable EL devices. Shape-ductile and self-healing EL displays have been offered in the last few decades. EL devices bear more than 100 cycles of failure–recovery actions. This helps to sustain the lifespan of the EL device, which further promotes sustainability and low-cost production. Outstanding stretchability (2500%) and high self-healing efficiency (96%) were attained by introducing reversible imine bonds [58]. This is the highest stretchable EL device with improved self-healing properties. The thermal properties of hydrogels are equally important for long-term stability [59].

**Figure 3:**
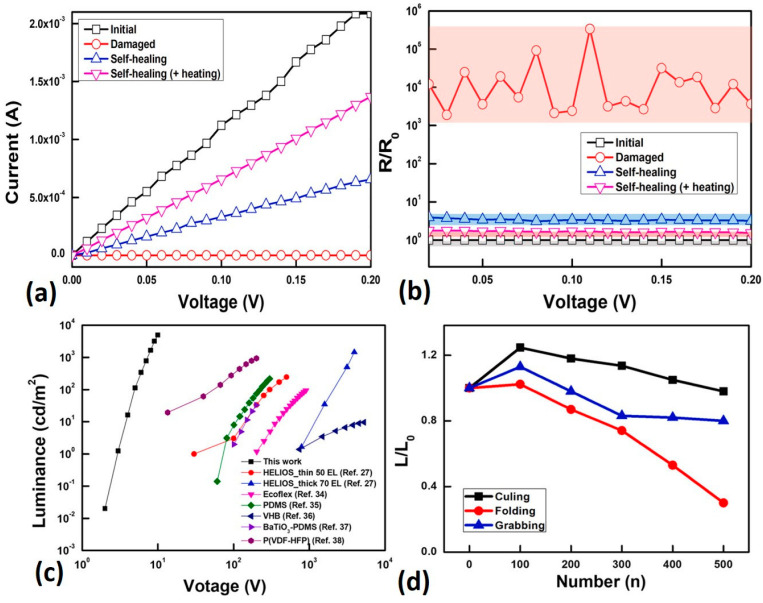
Electrical and luminescence properties of self-healable organic light-emitting devices. (**a**) Voltage vs. current diagram in which self-healing helps obtain better resistance against deformation. (**b**) Voltage vs. resistance curve showing the recovery of the crack after deformation. (**c**) Voltage vs. luminescence curve that shows the comparison of various types of materials with self-healing characteristics, indicating better luminescence behavior. (**d**) Luminescence ratios at various physicomechanical actions. Re-printed with permission; License Number: 5484060034281 [52]. Copyright ©2023, Journal of Nano energy, 2211-2855/Elsevier Ltd.

Figure 3 demonstrates the electrical and luminescence characteristics of self-healing materials for wearable applications.

In addition to this, a various number of self-healing polymeric materials have been reported for EL device fabrication, confirming the importance of being self-healable, stretchable, flexible, and having other unique properties for specified applications. Gao, L. et al. [60] described self-healing polymers that are able to completely restore dielectric properties against electrical treeing. The microcapsule approach helped to mend electrical failures against unfavorable conditions using polymer processing, which improved the lifetime of dielectric materials. Furthermore, a reversible cross-linkable PU has been demonstrated elsewhere [61].

Self-healing electroluminescence devices have diverse applications, such as in artificial skin, soft robotics, flexible electronics, wearable electronics, fashion clothing, actuators, different digital displays, and sensors. Electroluminescence devices have been combined into functional and flexible electronics as a light-emitting part. However, a long service life was not possible due damage occurred by mechanical strain. With self-healable hydrogels, such problems have been eliminated. Self-healable hydrogels are stretchable, stable for many cycles and can return to their original position after stretching.

### 3.2. Synthesis of Self-Healable Hydrogels

Self-healing hydrogels are next-generation materials for various applications. They can be synthesized using different methods depending on the compatibility and properties of the starting materials. Liu, Y. and S.h. Hsu [62] described the synthesis of self-healing hydrogels by incorporating nanomaterials into a dual -network hydrogel. They also mentioned that non-covalent interactions, hydrogen bonding, covalent interactions and dual networks play an important role in restoring the properties of self-healing hydrogels. Hierarchical dynamic cross-links with multiple hydrogen bonds have been developed to impart remarkable mechanical properties to hydrogels, which are characterized by extreme ductility, toughness under high real load and good fatigue resistance [63]. Simple mixing of polymers under certain conditions could also contribute to synthesized hydrogels [64]. An effective and stable inorganic optoelectronic film with a rigid epitaxial substrate could be mixed to a foreign flexible/soft substrate to compensate for mechanical properties such as stretchability and self-healing ability. The most common synthesis method of hydrogels for EL applications is cross-linking with various nanocomposite materials [65]. An alternative synthesis method is photopolymerization of aqueous solutions using visible lights [66]. The fundamental question in the synthesis of hydrogel materials is whether the hydrogel is fully functional in terms of flexibility, stretchability, mechanical strength, and durability or not. The composite materials should be compatible and must fulfil the above properties to blend together. In general, there is no set way to synthesis hydrogels that has been reported, but depending on the final application, each researcher can develop their own mechanisms, as long as the required properties are achieved. 

## 4. Hydrogels for EL Device Fabrication

**Figure 4:**
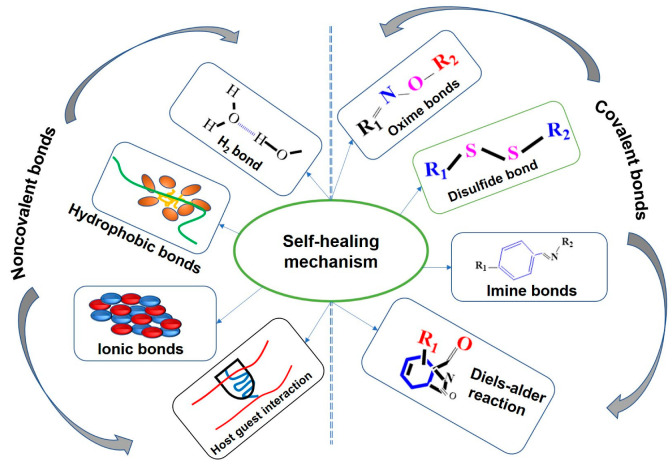
Self-healing mechanisms for hydrogel materials. Re-printed from an open access article under the terms of Creative Commons Attribution 4.0 International License of Ref. [70].

Hydrogels are of great interest due to their potential applications in flexible electronics, including electroluminescent materials. Recently, the molecular design of hydrogel materials with excellent physicomechanical properties has been reported [67,68]. Self-healing improved mechanical strength, greater flexibility, and ease of processing are the basic requirements of hydrogel materials for electroluminescence applications. This section reviews the different types of materials used to fabricate hydrogel materials for EL devices. Special attention is given to recent advances and typical properties of hydrogel materials. In addition, the critical challenges of hydrogel materials are also discussed. It is very difficult to achieve such diverse properties with a single polymer, and it is still difficult to obtain complex properties for flexible electronics by fabricating composites [69]. Self-healing is a mechanism in the formation of hydrogels. The self-healing mechanism is based on the formation of bonds. Bonds are responsible for the chemical and physical stability of hydrogel materials. The main bonds formed during self-healing are covalent and non-covalent bonds [70]. Figure 4 illustrates the different bonds formed during the formation of self-healing hydrogels.

As can be observed in Figure 4, basic self-healing mechanisms are classified into two broad categories, namely chemical/covalent bonding, and physical/non-covalent bonding. Chemical bonding includes the Diels–Alder reaction, imine bonds, disulfide bonds and oxime bonds, and they exhibit stronger but slower dynamic equilibrium when compared to that of physical bonds [71]. They have their own cross-linking behavior. For instance, oxime bonds are formed by the reaction between hydroxylamine and aldehyde groups [72]. On the other hand, self-healing hydrogels can be produced using physical cross-links, including host–guest interaction, ionic bonds, hydrophobic bonds, and hydrogen bonds. These bonds are less enduring and are more vulnerable to pH and temperature [71]. These bonding systems also follow different cross-linking approaches. For example, a hydrogen bond is formed because of the interaction of hydrogen atoms and electronegative atoms, such as nitrogen, oxygen and fluorine [71]. 

According to the report made by Devi VK, A. et al. [70], self-healing mechanisms can be divided into chemical cross-linking (covalent bonds) and physical cross-linking (non-covalent bonds). The name implies that physical cross-linking is considered mechanically weak compared to chemical cross-linking due to its weak intermolecular forces and reversible interactions. Chemical cross-linking, on the other hand, is based on the idea of immediate permanence, i.e., the variable buildup and degradation of apparatus occurs simultaneously and uninterruptedly throughout the reaction system. In chemical cross-linking, the networks could break down and re-form unconventionally. In addition, chemically cross-linked hydrogels are preferable for electroluminescence applications where the materials are subjected to the greater stresses because of their autonomous regeneration (self-healing) capability. Chemically cross-linked hydrogels are permanently durable, whereas physical cross-links are not [73]. In this section, some examples of chemically cross-linked hydrogels for electroluminescence applications are discussed. Bonding and cross-linking hydrogels have several advantages, including increased elasticity, decreased viscosity, increased insulating ability, increased glass-transition temperature, high strength and toughness, lower melting point, and increased chemical bonding [73]. Due these reasons, hydrogel formation is very critical for the final properties of the hydrogels formed. Therefore, it is very critical to differentiate and set the paarmeters before the syntehis of the hydrogel materials that determine the final proeprties of the hydrogel material.

### 4.1. Polyacrylic Acid-Based Hydrogels

**Figure 5:**
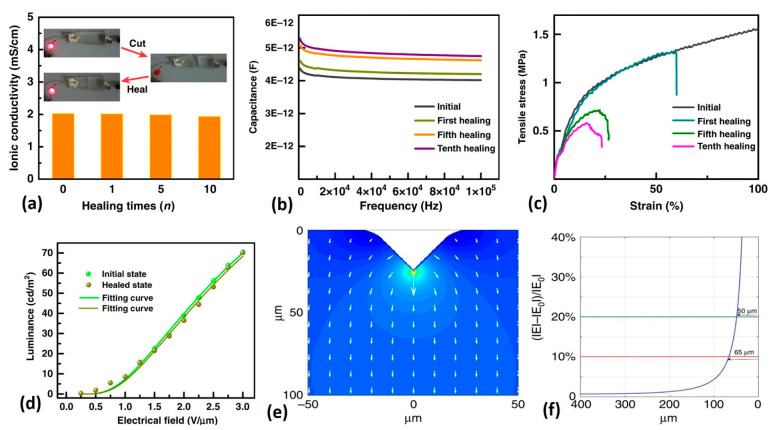
Self-healing and light-emitting performance of electroluminescence devices produced based on polyacrylic acid (PAA)-based hydrogels. (**a**) Ionic conductivity of PAA conductor after lots of cutting and healing times, showing almost full restoration of ionic conductivity after healing. (**b**) Dielectric capacitance after lots of healing and cutting times, which restored dielectric permittivity and capacitance and revealed that light-emitting intensity remains constant after healing. The capacitance of the dielectric layer remained constant after various healing cycles with only a 16.8% increase in capacitance at 1000 Hz. (**c**) Mechanical performance of the EL device with the same healing–cutting times shows restoration of 537 kPa in tensile strength and 2.4 MPa in Young’s modulus. (**d**) Voltage-luminescence performance of the EL device at the very beginning states showing high mechanical strength and good flexibility. (**e**) Distribution of the electrical field across the pigment layer (magnified image), showing that the electrical field variation dispersed within the space span. (**f**) Variation in the electric field. Re-printed from an open- access article under the terms of Creative Commons Attribution 4.0 International License of Ref. [43].

Polyacrylic acid (PAA) hydrogels have been widely used to fabricate electroluminescence (EL) devices, owing to their unique characteristics, including self-healing ability and outstanding physicochemical properties [43]. Figure 5 demonstrates the self-healing and light-emitting performance of electroluminescence devices using polyacrylic-acid-based hydrogels.

In situ polymerization of PAA-based hydrogels has been synthesized, obtaining a deep-red-emitting supermolecular hydrogel (λ_em,max =_ 651 nm, Φ_F_ = 0.17) [74] Furthermore, polyacrylic acid played a very important role in the formation of gelation. Again, PAA has been combined with silica hydrogel nanofibers and showed excellent photoluminescence performance [75]. However, cross-linking was moderate, which is limited in terms of long-term stability. Indeed, double-network PPA-based hydrogels could be possible when combined with polyvinyl alcohol (PVA) [76]. This improves the strength, as well as increases the stability of the hydrogel. Alternatively, luminescent-responsive hydrogels in humid or aqueous environments can be realized using PAA composites [77]. These multi-stimuli-responsive hydrogels were fabricated by combining PAA with a network of polyacrylamide into lanthanide ions and carbon dots. Furthermore, lanthanide-based hydrogels with PAA as an organic network was produced, which was able to obtain various luminescence colors, in addition to its robust self-healing properties [78]. PAA-based hydrogels are highly stimuli-responsive [79]. This kind of behavior helps to mitigate different stimuli including pH, humidity, temperature, and other environment-based stimuli that might fluctuate during use. PAA-based hydrogels are preferred whenever environmental changes are expected and the hydrogels are strong and robust in its application.

### 4.2. PMA-Based Hydrogels

Poly (methacrylic acid) (PMA) hydrogels have been prepared using bulk polymerization with very high mechanical properties [80]. As a result of this, both hydrophobic and hydrogen cross-linking existed simultaneously, which helped the hydrogels to possess excellent softness and stiffness. This shows the feasibility of using PMA -based hydrogels for EL device fabrication. In addition, poly (methacrylic acid) hydrogel capsules are used for effective luminescence-based applications [81]. The composite materials with poly (methacrylic acid) with blue phosphor BaMgAl_10_O_17_:Eu^2+^ showed a high color-rendering index of 87.0. In most cases, the electrode and the dielectric materials for EL applications are not single molecules or polymers; they are usually combined with other polymeric materials to compensate for the critical properties of the EL device. For instance, the conjugated poly[(2-methoxy-5-((2-ethylhexyl)oxy)-1,4-phenylene)vinylene] (MEH−PPV) with a side chain luminescent polymer and an alkoxy(trifluoromethyl) stilbene-substituted PMA derivative (CF_3_−PMA) have been demonstrated to develop light-emitting diodes [82]. When the mixing ratio of the two polymers varies, the luminescent colors also vary, i.e., there is a possibility of the formation of two spectral emissions. Another intriguing fabrication of electroluminescence using PMA-based hydrogel is reported in Ref. [83], where a composite of PMA and CuInS_2_ quantum dots composites was developed [84]. The long-term stability of EL devices can be compensated by such composite means. Surface engineering and surface coating have potential advantages to overcome long-term stability problems. In addition, Zhu, Q. et al. [77] combined PMA with lanthanides and carbon dots to induce changes due to optical and mechanical actions that act up on it. This type of hydrogel helps to withstand multiple stimuli, including pH, organic vapors, transition-metal ions and temperature. Correspondingly, the composite is not only chemically responsive, but it is also capable of stretching with a fracture strain of 400%. Indeed, the advantages of hydrogel-based light-emitting devices are two-fold; it responds both mechanically and chemically to combat long-term stability, which is known to be a problem in conventional light-emitting diodes. Another very important discovery in the synthesis of PMA-based hydrogels for luminescence materials is the making composite materials with m-phenylenediamine and doping with graphene oxide and carboxymethyl chitosan to improve the structure [85]. Furthermore, the mechanical and light absorption capabilities of the hydrogel confirmed that the materials possess acceptable luminescence characteristics. In general, PMA and its composite-based hydrogels have shown tolerable characteristics with excellent self-healable properties, which are required for the production of stable electroluminescence devices.

### 4.3. Silk-Fibroin-Based Hydrogels

**Figure 6:**
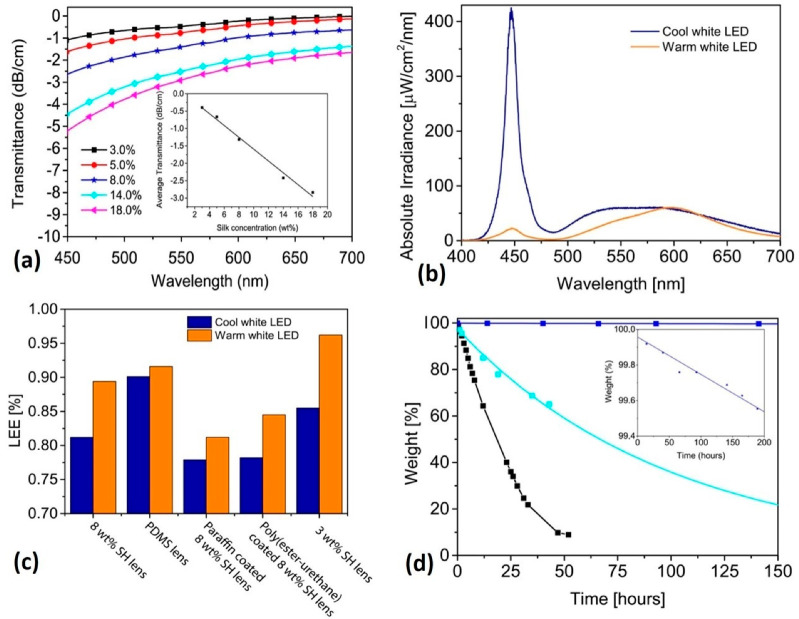
Optical and light-extraction stability of silk fibroin hydrogel. (**a**) Optical transmittance of silk fibroin hydrogel in visible spectrum at various fibroin protein concentrations, showing that as the fibroin concentration increases, the hydrogel becomes yellowish in color. (**b**) Absolute irradiance of cool and warm white LEDs shows a high peak around 450 nm (blue and orange peaks). (**c**) Light-extraction efficiency of the silk fibroin hydrogel without top coating and poly(dimethylsiloxane) (PDMS) lens on cool and warm white LEDs showing that a decrease in silk concentration resulted in preferable integration with the warm white LEDs. (**d**) Loss in weight of silk hydrogel, showing rapid weight loss after 24 hrs. The weight loss is due to the evaporation occurred during the change of the temperature of the environment. Re-printed from an open access article under the terms of Creative Commons Attribution 4.0 International License of Ref. [89].

Silkworms are a natural source of silk, which is mainly composed of silk fibroin and sericin [86]. Within these components, 75% of silk is silk fibroin [87]. Correspondingly, silk fibroins are promising candidates for the preparation of hydrogel materials for several reasons. Among these, high water retention, self-healing ability and biocompatibility are some of them [88]. Furthermore, owing to the aforementioned properties, silk fibroin could be an effective entrant for the preparation of hydrogels for EL applications. Melikov, R. et al. [89] described light-extraction efficiency over 0.95 in warm white LEDs using fibroin lenses. However, the optical properties of the lenses could be affected by silk protein [90], to which special attention must be given. Indeed, the functional characteristics of silk fibroin led researchers to develop an EL device out of it [91]. The physical rigidity of the current materials used in the construction of EL devices forced researchers to focus on more flexible, lightweight and biocompatible materials, and silk fibroin is one of the prefect candidates. Compared to electroluminescent (EL) cells that are currently available, which need very high operating voltages but are easily adaptable to vast area formats, light-emitting diodes (LEDs) are fundamentally small in size, have low voltage, and are relatively high-current-density devices. Within this principle, silk fibroin hydrogels are very efficient in obtaining LEDs for different applications. Figure 6 shows the LED performance of hydrogels based on silk fibroin.

Flexile electronics, such as electroluminescence devices, serve as wearable electronics. During such applications, flexible electronics face several unconventional interfaces, such as bending, flexing, tension and other compressional forces. This makes it difficult to use conventional materials for such applications as they cannot confront such actions. Silk is a biological polymer and can offer very new prospects for flexible electronics because silk-based fibroin has the capability to self-heal. In addition, silk offers superb mechanical, electrical and optical properties [92]. This makes silk fibroin hydrogels a perfect contender for flexible electronics such as light-emitting diodes. Functional hydrogels from silk fibroin possess high strength; double-network structure; interpenetrating network structure, which can cross-link at low temperatures and can be easily injected; capability to self-heal; excellent adhesion; high conductivity; excellent environmental stimuli; and is easily processable, such as 3D printability [86]. All the above-mentioned properties of silk-fibroin-based hydrogels could improve the service life of fabricated electroluminescence devices. Self-healable characteristics of silk fibroin hydrogels play an important role in its applications for flexible electronics such as light-emitting diodes [93,94,95,96,97,98,99,100]. As can be observed in different literature findings, the dynamic chemical bond method helped to prepare self-healing hydrogels from silk fibroin, which refers to the incorporation of amide and catechol groups and, consequently, the generation of dynamic diol-borate easter bonds [86]. Hydrogels from silk fibroin are not only self-healing but also electrically conductive [101]. Hence, when silk fibroin is combined with conductive polymers such as polypyrrole, the composite hydrogel exhibits electrical conductivity in the range of 0.8 ± 0.2 (1.0 ± 0.3) × 10^−3^ S.cm^−1^. Consequently, electrical conductivity plays an important role in constructing electrode materials for EL device fabrication.

In general, the following statements can be made:

1. Tough materials such cellulose nanocrystals could be incorporated in order to improve the mechanical stability of silk fibroin hydrogels [100];
2. To improve the stretchability and compressibility of hydrogels, polymers such as polyacrylamide, graphene oxide and poly(3,4-ethylenedioxythiophene): poly(styrenesulfonate) could be mixed with silk fibroin [99];
3. Silk fibroin could be chemically modified using photo-cross-linking with glycidyl methacrylate to improve the tensile properties and environmental stability [98];
4. To improve the rheological and ion-conductive properties of silk fibroin, silk fibroin can be injected with xanthan gum with the help of cross-linkers [97];
5. Hydrogels for EL applications from silk fibroin are eco-friendly [102];
6. To obtain real-time self-healing hydrogels, silk fibroin has been combined with tannic acid [103];
7. To enhance electrical conductivity, silk fibroin can be combined with highly conductive materials, such as graphene [104,105], to obtain efficient EL devices; and
8. Among the list of hydrogels for electroluminescence materials, hydrogels based on silk fibroin is one of the most researched one.

One of the most important required properties for EL applications is transparency. In this regard, silk fibroin hydrogels fulfil this fundamental property. Hu, F., N. Lin and X. Liu [106] stated a ca. 90% optical transparency of silk fibroin is very critical for optical applications. The functionality of silk is now beyond wearing applications. Silk fibroin hydrogels possess optical transparency with excellent and tunable mechanical strength [107]. Silk fibroin also has the capability to serve as an electrode, as well as a dielectric material in the fabrication of electroluminescence devices. For instance, Capelli, R. et al. [108] demonstrated the use of silk fibroin as a thin-film dielectric separator between the two electrodes. The silk-fibroin-based optoelectrical element provides an equivalent efficiency with organic transistors by providing a charge mobility of 10^−2^ cm^2^/V s and an on/off ratio of ten thousand. When cross-linked with, for instance, physical cross-linking including hydrogen bonds [109,110], silk-based hydrogels provide high mechanical performance, high stretchability and self-healing properties, which are required to construct self-healable hydrogel-based EL devices. This silk-protein-based hydrogel with high biocompatibility and strength diversifies strong hydrogels and shows significant promise as a potential candidate for light-emitting diodes in the electroluminescence industry.

### 4.4. Conductive Hydrogels 

**Figure 7:**
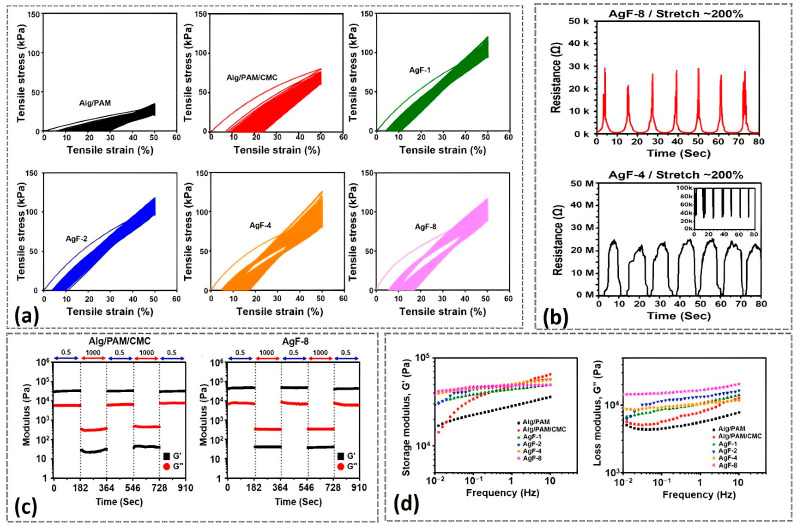
Mechanical, rheological, and electrical characteristics of CMC-based hydrogels. (**a**) Stress–strain curves of CMC-based hydrogels. (**b**) Resistance of CMC-based hydrogels at 200% stretch percentage against time. (**c**) Modulus vs. time curve for CMC-based hydrogels. (**d**) Oscillation frequency sweep curve of CMC-based hydrogels. Re-printed from an open access article under the terms of Creative Commons Attribution 4.0 International License of Ref. [121].

Hydrogels made of flexible conductive polymer are gaining popularity as electrode materials. Because conductive polymer hydrogels offer some benefits, including biocompatibility, high conductivity, 3D nanostructure, solvated surface, and expanded interface, electrochemical biosensors with these materials have been created. Electroluminescence devices consist of two electrodes separated by a dielectric layer and a luminescence layer. The use of a conductive hydrogel is therefore to act as a lower electrode and counter electrode. The lower electrode and counter electrode can be deposited on a flexible substrate, depending of the deposition principle. Functional polymers play a very crucial role in the construction of functional and smart materials [111,112,113]. Functional polymers are incorporated into compatible materials to create conductive hydrogels [101]. Traditional conductive hydrogels often do not have self-healing capabilities, but they could be useful in applications for smart electronics [114]. In addition, conductive hydrogels are frequently utilized in a variety of applications, including tissue engineering, flexible and implantable bioelectronics, and artificial skin [115]. For this reason, a new type of hydrogels called electrically conductive hydrogels (ECHs), which combine a hydrophilic matrix with different electric-conducting fillers, are required [116]. The authors showed an excellent property which merges electrical conductivity and self-healing characteristics. They also reported that the preparation of conductive hydrogels, employing different mechanisms such as radical polymerization, physical adsorption, chemical cross-linking and so on. A conductive hydrogel is a type of polymer material with a wide range of potential uses and a number of unique characteristics, such as high toughness, self-recoverability, high electrical conductivity, greater transparency, excellent freezing resistance, stimuli responsiveness, high stretchability, good self-healing and excellent strain sensitivity [117]. An electrically conductive silver–polyacrylamide–alginate hydrogel composite for soft electronics has been reported elsewhere [118]. However, there are still significant obstacles to overcome before conductive hydrogels can be concurrently combined to obtain exceptional mechanical strength and conductivity without losing their versatility [119]. Hydrogel composites help to improve compliance and recoverability from deformation. Conductive hydrogels without self-healing properties might not be appropriate for the fabrication of EL devices. The use of conductive hydrogels with self-healing properties for EL applications have been reported [18,120]. In addition, electrical conductivity can be combined with stretchability and achieved at the same time when self-healable conductive hydrogels are used for luminescence materials. Rheological, mechanical and electrical performances are paramount for conductive hydrogels to be used as light-emitting diodes. For instance, Park, K. et al. [121] demonstrated conductive hydrogels based on carboxymethylcellulose (CMC). Figure 7 shows the various performance of luminescence materials based on conductive CMC hydrogels.

One of the critical advantages of using hydrogels for luminescence materials is their resistance against mechanical damage or their self-healing behavior. The results from Figure 7a showed that CMC-based hydrogels could tolerate cyclic strains more than 100 times, which is very important for materials exposed for repeated stretchability and bendability during service. Furthermore, the additions of conductive hydrogels based on CMC improved the modulus properties (Figure 7c,d). The endurance of electrical conductivity against stretchability (Figure 7b) showed that CMC-based hydrogels can be demonstrated successfully for the fabrication of light-emitting diodes under mechanical deformations.

In addition to this, various numbers of conductive hydrogels materials have been reported for EL device fabrication, confirming the importance of being self-healing and stretchable and having ionic conductivity, among other unique properties, for the specified applications. Electrically conductive hydrogels must not only exhibit conductivity but must also be insoluble in aqueous electrolyte solutions [122]. Table 1 illustrates the comparisons of different conductive hydrogels for electroluminescence applications.

Biocompatible and intrinsically conductive polymers with various properties can be mixed to form composites. In such cases, conductive polymers provide electrical conductivity, while other polymers provide flexibility, stretchability, self-healing ability, and high mechanical strength. With such uncompromised properties, self-healing electrode materials can be prepared for EL applications. Among the investigated hydrogels for EL applications, conductive hydrogels are among the most studied for EL applications (See Table 1).

Conductive hydrogels are promising constituents for electroluminescence applications. To satisfy the diverse requirements of EL devices, namely flexibility, stretchability, conductivity, self-healing ability, and ductility, using conductive hydrogels is the ideal solution. However, due to the hydrophobic nature of conductive materials, process ability still remains a challenge [130]. Sometimes, EL devices are exposed to biaxial forces; therefore, ionic conductors based on liquid crystal have been reported to compensate for this problem [131]. Furthermore, conductive hydrogels have excellent properties for electroluminescence applications, including good elasticity, excellent toughness, high self-healing ability, and acceptable mechanical strength [116]. These properties are highly required for self-healing EL device fabrication in which chemical cross-linking, which provides covalent bonding to the hydrogel, plays an important role for such properties. Covalent bonds (permanent cross-linking) are better in terms of performance than non-covalent bond (physical bonds). In this regard, conductive hydrogels are most often fabricated using covalent bonds [132,133,134,135]. Conductive material based on silver-grid/PEDOT:PSS has been successfully printed on a flexible epoxy film and obtained with stable LEDs up to 30,000 bending cycles [136]. Properties such as self-healing, stretchability and excellent mechanical properties are realized more when chemical cross-linking occurs. These properties are very important for self-healable electroluminescence device fabrication.

### 4.5. MXene-Based Hydrogels

Soft electronics that are mechanically malleable and pliable are pushing the boundaries of stiff and conventional electronics devices. In material science, MXene is a class of two-dimensional inorganic compounds that consist of automatically thin layers of transition metal carbides, nitrides, or carbon nitrides. MXene-based hydrogels are highly conductive hydrogels with excellent electromechanical properties [137], where conductivity plays a significant role in the diverse applications of this hydrogel [138]. Self-healing and biocompatible MXene-based hydrogels for multifunctional applications have been reported in Ref. [139]. The biocompatible conductive hydrogel was a prime choice for wearable multifunctional sensors for strain sensing, vocal sensing, signature detection and Morse code transmission because of its sensitivity (gauge factor of 2.16), self-healing (within one s), recognition and adherence. These kinds of hydrogels are highly conductive and can be used in multifunctional applications, including EL device fabrication, as electrode materials. Current sources for EL devices can be varied. Sun, Z. et al. [140] verified flexible alternating-current electroluminescence devices using multilayer MXene/cellulose nanofibril composite film. The composite film provided efficient light emitting with an output voltage of approximately 90 V at a frequency of 2 Hz. With such composite film, the self-powered principle was employed, which avoids the use of an extra power source. This makes it easier to use MXene-based hydrogels for wearable electronic applications. MXene-based hydrogels are also highly stretchable and possess self-healable properties [141], which is fundamental to EL device fabrication.

MXene-based hydrogels are found to be highly conductive, can be self-powered, self-healable and highly stretchable, and its enhanced performances makes them the ideal candidate for wider applications [142]. Therefore, it is recommendable to use MXene-based hydrogels when such critical characteristics are required during the fabrication of soft electronics, including electroluminescence device fabrication.

### 4.6. Polyvinyl-Alcohol-Based Hydrogels

Its excellent mechanical properties, hydrophilicity, chemical and physical stability, film formation capability, biocompatibility nature and flexibility make polyvinyl alcohol (PVA) an excellent choice for the fabrication of hydrogel materials for luminescence applications [143]. PVA can be incorporated into several polymers and can create excellent luminescence properties. Liu, Y. et al. [144] claimed PVA-based hydrogels showed excellent mechanical and transparency properties when cross-linked with the 1/ethyl-3-methylimidazolium acetate/water (EMImAC/H_2_O) compound. The outstanding transparency property is an open path for the fabrication of light-emitting diodes with better stability properties. PVA not only has excellent mechanical properties but also good stretchability and conductivity [145]. From these properties, PVA-based hydrogels can possess up to 1100% stretchability and 1.34 kΩ cm. Such kinds of hydrogels can serve in producing EL devices with excellent self-healable properties. Due to its biocompatibility, PVA can form excellent composite materials with other polymers, including soluble Cu and Au nanocellulose, using the casting method [146]. The composite exhibited a color-rendering index of 86 with Commission Internationale de l’Eclairage (CIE) coordinates of 0.33 and 0.35. The LEDs tuned from 5582 to 9490 K. This characteristic nature has shown that PVA metal composite have strong luminescent ability. These kinds of formulations are most common in hydrogel synthesis. This is because electroluminescent devices require several combinations of physicomechanical properties.

EL devices fabricated using PVA-based hydrogels showed strong self-healing properties [147]. This improves the stability of EL devices against mechanical deformations. EL devices require not only self-healing ability, but also electrical conductivity. In the structure of EL devices, there are upper and lower electrodes to connect with AC or DC voltage sources. Dispenza, C. et al. [148] introduced novel nanocomposites of polyaniline (PANI) and obtained functional hydrogels with better transparency and environmental sensitivity. Hydrogels based on PVA also have the capability to be printed on textiles materials [149], which means PVA-based hydrogels are more compatible for textile substrates. Table 2 summarizes the fabrication, composites and other detailed results of EL devices fabricated from PVA-based hydrogels.

PVA-based hydrogels are one of the most researched composite hydrogels. They have been widely explored because of their biocompatible nature, superior water absorbing capability, excellent chemical stability and nontoxic nature. Therefore, it is not a surprise that PVA hydrogels are the ideal candidate for the manufacturing of EL devices. PVA also has excellent transparency properties and has the ability to mix with highly stretchable polymers, including conductive polymers, to compensate for its drawbacks. PVA-based composite polymers show excellent physicomechanical and electrical properties, which can be used as good electroluminescence materials. Furthermore, PVA-based hydrogels can comprise many properties, such as flexibility, stretchability, deformability and compatibility. In general, PVA-based hydrogels are one of the candidates for the manufacturing of electroluminescence devices. Therefore, PVA-based hydrogels continue to be used in EL fabrication.

## 5. Challenges of Hydrogel-Based Materials for Electroluminescence

Although developments in the use of hydrogel-based self-healing materials show promising progress, there are still challenges in this area. There are several problems in the fabrication of hydrogel-based electroluminescent devices. Many studies are underway to solve these problems. In this review, we identified the following challenges in using hydrogel-based materials to fabricate EL devices. First, EL materials should be hydrophilic, self-healing and flexible. However, the hydrophilic properties of the polymer need to be further improved because some of the conductive hydrogels are hydrophobic [130], which could complicate the process. Another challenge of hydrogels is their low tensile strength and environmental stability [98]. Therefore, maintaining and improving the mechanical strength of the processed material has become an important issue for 3D hydrogel structures. The main disadvantage of hydrogels is that they have low adhesion and require a secondary material to bond and secure cross-linking with the substrate materials [154]. Therefore, a matrix polymer is required to increase adhesion strength. In addition, to increase stability and avoid mechanical problems, covalent bonds are required in gel formation instead of non-covalent bonds, such as hydrogen bonds. Hydrogels are also difficult to combine, respond to stimuli, are self-healing, biocompatible, and conform to patterns [139]. Therefore, it is important to note that some of the materials with these properties must be considered when developing EL devices based on hydrogels. The large number of cross-links in the formation of hydrogels, including chemical and physical cross-links, results in fragile/brittle hydrogels due to the restrained expansion of polymer chains and high strain in weak chains within heterogeneous networks. 

Another drawback of hydrogel-based electroluminescent devices is their sensitivity to the environmental conditions, such as pH and oxidation [148]. Because EL devices are always exposed to such conditions, researchers need to consider these aspects when developing hydrogel-based EL devices. On the other hand, the lack of dynamic properties and the structural complexity of hydrogels also limit their application in the fabrication of EL devices [68]. These challenges force researchers to focus on the development of hydrogels that can fill such gaps. Although electroluminescence hydrogels exhibit a change in geometric shape when exposed to light, they suffer from relatively low deformation [155]. Therefore, the occurrence of such problems limits the use of hydrogels in the fabrication of electroluminescent devices on a larger scale, and thus future researchers are still challenged with developing fully functional hydrogels for the fabrication of EL devices. This review article shows that research on hydrogel materials for EL devices is still a hot topic and much research is still being conducted. Because of this, there is reasonable hope that all the above problems could be solved in the near future and that we will see highly effective hydrogel materials for the sustainable fabrication of electroluminescent materials.

## 6. Future Outlooks and Conclusions

In this review article, we have highlighted the importance and properties of self-healing materials for electroluminescent device fabrication. Materials with different self-healing capabilities can be synthesized, characterized and explored according to the requirements for electroluminescent device fabrication. It is essential to note that properties such as high ductility, cyclic stability, conductivity, flexibility, transparency and ductility should be considered and are very important for EL device fabrication. In this regard, conductive, fibroin-based and acrylic-based hydrogels are the most promising candidates for such applications. Although high conductivity and ductility are highly desirable for many EL materials, the hydrophobic nature of conductive materials must be considered during processing. However, numerous problems have been identified, in which hydrogel-based materials are not yet presented well in the EL manufacturing market. The synthesis of hydrogels involves the formation of chemical and physical bonds. These bonds can be formed by various mechanisms, such as mixing and casting solutions, polymerization, irradiation, the formation of free radicals and the formation of cross-linking networks.

Researchers have made great efforts to develop hydrogel-based electroluminescent devices that can convert electrical energy into light energy, and the introduction of hydrogels in this field has driven the development of EL devices. Electroluminescent devices made from hydrogels show improved performance, although there are still problems, such as brittleness, poor mechanical strength, low stability and low adhesion. Various strategies have been employed to address these problems, such as incorporating matrix polymers and using appropriate cross-linkers. The use of matrix polymers significantly improves the mechanical properties, flexibility, stability and self-healing properties of hydrogels. This review provides a few insights into hydrogel materials for EL applications, some challenges, and the future directions.

## Figures and Tables

**Table 1:** Comparison of conductivity-based hydrogel materials for electroluminescence applications.

Conductive Materials	EL Materials	Dielectrics	Performances	Ref.
Polyacrylamide hydrogels	phosphor	acrylic elastomer	luminance when stretched up to an area strain of 1500%, self-healing	[18]
Polyacrylamide with Li^+^ ion	phosphor	-	1600% extension, tension 0.22 MPa and toughness 2.2 MJ/m^3^, self-healing	[16]
potassium iodide and glycerol	ZnS: Cu	polyvinylidene fluoride (PVDF)	even light emission at 140% stretching along with bending, rolling, and twisting	[123]
poly (acrylic acid) + PEDOT: BCNF ^a^	-	-	showed ultra-stretchability of 2850% and speedy independent self-healing	[124]
Zwitterionic Nanocomposite	-	-	stretchability over 1000% strain, tensile strength up to 0.61 MPa	[125]
BMMIm [TFSI-based ^b^	ZnS: PDMS	acrylic elastomer	stretching ability up to 500%, self-healing	[126]
MXene and AA-based ^c^	ZnS: Cu with PDMS ^d^	-	ultra-elasticity (1000% to 3200%), self-healing	[127]
PAM-PVA Hydrogel	phosphor	silicone elastomer	highly stretchable (over 1500%)	[128]
PAAm hydrogel + PEDOT: PSS	ZnS	poly (2-hydroxyethyl acrylate)	extremely high stretchability and elastomeric properties	[129]
Polydopamine-doped PPy	-	-	high tensile strength up to 120 kPa tough and stretchable.	[130]

^a^ Sulfonated bacterial cellulose nanofiber; ^b^ IL 1-butyl-2,3-dimethylimidazolium bis(trifluoromethylsulfonyl) amine; ^c^ acrylic acid; ^d^ polydimethylsiloxane.

**Table 2:** Comparison of PVA-based hydrogel materials for electroluminescence applications.

Composite	Fabrication Methods	Performances	Ref.
Polyacrylamide	free radical polymerization	excellent stability and EL properties, lights up to 50 LEDs (ZnS:Cu phosphor)	[128]
Phytic acid	one freeze–thaw cycle	conductivity of 1.34 kΩ and 95% optical transparence	[145]
Polyacrylamide	aqueous synthesis	high color-rendering index of 92.1 (R9 = 92.0) was obtained	[150]
Metal nanoclusters	aqueous polymerization	color-rendering index (CRI) of 86 with CIE color coordinate of (0.33, 0.35)	[146]
Pectin	aqueous polymerization	luminescent intensity at 473 nm and 617 nm (I473/I617)	[151]
Polyaniline (PANI)	chemical oxidative polymerization	fluorescence is observed from PANI	[148]
Polyurethane (PU)	mechanical deposition	electroluminescence intensity at 514 nm	[52]
Polyacrylamide	injection	highly optical transparency	[16]
Poly (acrylic acid)	cross-linking via H_2_ bonding	excellent Conductivity	[152]
Carbon dots	immobilization	bright blue fluorescence with the quantum yield of 0.35	[153]

## Data Availability

Not applicable.
